# Metamorphoses of Lyme disease spirochetes: phenomenon of *Borrelia* persisters

**DOI:** 10.1186/s13071-019-3495-7

**Published:** 2019-05-16

**Authors:** Natalie Rudenko, Maryna Golovchenko, Katerina Kybicova, Marie Vancova

**Affiliations:** 1 Biology Centre CAS, Institute of Parasitology, Branisovska 31, 37005 Ceske Budejovice, Czech Republic; 2 National Institute of Public Health, Srobarova 48, 100 42 Prague 10, Czech Republic

**Keywords:** *Borrelia burgdorferi*, Persisters, Dormant forms, Round bodies, Biofilm, Lyme disease, Persistent infection, Antibiotic treatment

## Abstract

The survival of spirochetes from the *Borrelia burgdorferi* (*sensu lato*) complex in a hostile environment is achieved by the regulation of differential gene expression in response to changes in temperature, salts, nutrient content, acidity fluctuation, multiple host or vector dependent factors, and leads to the formation of dormant subpopulations of cells. From the other side, alterations in the level of gene expression in response to antibiotic pressure leads to the establishment of a persisters subpopulation. Both subpopulations represent the cells in different physiological states. “Dormancy” and “persistence” do share some similarities, e.g. both represent cells with low metabolic activity that can exist for extended periods without replication, both constitute populations with different gene expression profiles and both differ significantly from replicating forms of spirochetes. Persisters are elusive, present in low numbers, morphologically heterogeneous, multi-drug-tolerant cells that can change with the environment. The definition of “persisters” substituted the originally-used term “survivors”, referring to the small bacterial population of *Staphylococcus* that survived killing by penicillin. The phenomenon of persisters is present in almost all bacterial species; however, the reasons why *Borrelia* persisters form are poorly understood. Persisters can adopt varying sizes and shapes, changing from well-known forms to altered morphologies. They are capable of forming round bodies, L-form bacteria, microcolonies or biofilms-like aggregates, which remarkably change the response of *Borrelia* to hostile environments. Persisters remain viable despite aggressive antibiotic challenge and are able to reversibly convert into motile forms in a favorable growth environment. Persisters are present in significant numbers in biofilms, which has led to the explanation of biofilm tolerance to antibiotics. Considering that biofilms are associated with numerous chronic diseases through their resilient presence in the human body, it is not surprising that interest in persisting cells has consequently accelerated. Certain diseases caused by pathogenic bacteria (e.g. tuberculosis, syphilis or leprosy) are commonly chronic in nature and often recur despite antibiotic treatment. Three decades of basic and clinical research have not yet provided a definite answer to the question: is there a connection between persisting spirochetes and recurrence of Lyme disease in patients?

## Background

Lyme disease (LD) is a multisystem disorder caused by certain species of spirochetes from the *Borrelia burgdorferi* (*sensu lato*) complex. Since all *Borrelia* species are host-propagated bacteria that move between a vertebrate host and tick vector, the spirochetes have developed strategies to sense and survive in these diverse environments [1–4]. Survival is achieved by altering the level of gene expression in response to changes in temperature, pH, salts, nutrient content, multiple host and vector dependent factors [5–10]. Nutrients, especially carbon sources, and/or their metabolic by-products seem to provide regulatory and chemotactic signals that guide the spirochete as it moves between hosts and vectors. The spirochetes that are exposed to antibiotic treatment are able to survive by regulation of the differential expression of genes involved in spirochete pathogenicity and the mechanism of persister formation [11–13]. However, the change in gene expression level is not the only route to spirochete survival. Signals that *Borrelia* receives from hostile environments evoke morphological alterations that keep the pathogen alive and induce the production of atypical forms or persisters that are refractory to elimination. The formation of persisters *in vitro* and *in vivo* is a reversible process that establishes the basis for disease recurrence when the hostile pressure drops [8, 14–18].

The successful persistence of spirochetes within the host depends on evading the host’s immune system, e.g. hiding of spirochetes within the extracellular matrix rather than using the host tissues for reproduction or growth [19–21]. Multiple experimental animal studies have shown that *Borrelia* frequently establishes persistent infection in diverse vertebrate hosts including laboratory mice of C3H/HeJ, BALB/cByJ, C3H/HeNCrlBR, C3H-*scid*, SJL or B6 Myd88−/− genotypes [17, 22–28], white-footed mice *Peromyscus leucopus* [29, 30], rats [31], hamsters [32, 33], guinea pigs [34, 35], gerbils [36], dogs [37–39], horses [40–42] and non-human primates [43–49]. Clinical evidence extends this paradigm to humans including those who underwent antibiotics treatment [50–85].

The hosts’ immune responses to LD spirochete, pharmacokinetic and pharmacodynamic parameters of antibiotic treatment in animals and humans, infectious dose and the route of infection are not the same [86]. It is clear that results obtained on laboratory animal models might not be simply applied to explain the phenomenon of persisters in human LD. However, the extended examples of diverse strategies used by LD spirochete in its competition for survival, i.e. active immune suppression, induction of immune tolerance, phase and antigenic variation, intracellular seclusion, or incursion into immune privileged sites, provide strong evidence of the capability of spirochetes to persist in vertebrate hosts [4, 87].

## Facing antibiotic challenge

Frequent failures to respond to antibiotic treatment have recently become a serious public health problem. The common explanation of such cases is the emergence of a superinfection as a result of the development of bacterial resistance. Bacteria are able to develop resistance to the majority of clinically used antibiotics and the extended emergence of multi-drug-resistant pathogens is secured by the wide use of antibiotics in daily life by the general population, in hospitals, in agriculture or farming. There is no doubt that resistance is the main culprit of antibiotic treatment failures; however, less obvious reasons must be taken into consideration as well. One of these reasons is a phenomenon known as bacterial persistence, the case when bacteria survive the killing action of antibiotics by changing its physiological state.

Lyme disease is an infectious disease that can usually be successfully cured by antibiotic therapy at the very early stages of infection, targeting the replicative form of the spirochete [88–90]. Antibiotic treatment of microbial populations in general and *B. burgdorferi* (*s.l.*) in particular, results in biphasic killing [16, 18], eliminating the growing bacteria and inducing the appearance of a subpopulation of multi-drug tolerant cells, persisters [91, 92]. When the antibiotic concentration exceeds a certain threshold, only persister cells survive [18]. Persisters are tolerant to antibiotics without having acquired resistance to them through genetic modification [93]. Persistence is a non-inheritable feature. This discriminates persistent cells from resistant mutants, which exhibit stable, inheritable drug insensitivity [94]. A decrease of antibiotic pressure leads to the rise of a cell population that is as susceptible to antibiotics as the original population. A raised cell population called “reverters” constitutes a very small proportion of persisters that could revert to replicating forms and cause relapse or chronic infection [18, 95].

Persistence, as the response to hostile challenge, is an essential strategy for the complex life-cycle of *Borrelia* in both vector ticks and reservoir hosts, and likely applies to regular vertebrate hosts as well as to incidental ones such as humans. The ability of LD spirochetes to transform into persisters in response to antibiotic treatment explains the surprising resistance of chronic infection to therapy with antibiotics that are effective in the elimination of replicative *Borrelia in vitro* [18].

## Different stressors: diverse pleomorphic forms

In addition to the well-known flat-wave forms of *B. burgdorferi* (*s.l.*) spirochetes, the existence of non-motile atypical morphologies such as looped or ring shaped forms, blebs, round bodies (RB) and cell wall deficient forms, spirochete colonies or biofilm aggregates have been described [14, 95–105]. The generalized definition of “round bodies” also includes coccoid-, globular- or spherical bodies, granules, cysts, sphaeroplasts or vesicles; all of these are viable reproductive propagules whose formation is induced by hostile environmental conditions [106]. The morphological changes of motile *B. burgdorferi* (*s.l.*) spirochetes are observed *in vitro* in response to incubation with water or serum starvation, a change of media composition, gas composition, oxidative stress, pH or temperature fluctuation, media acidity-alkalinity fluctuation, concentrations of salts, sugars or other organic compounds, or as a response to antibiotic treatment [7, 14, 100, 104–108]. The formation of RBs in culture is induced by aging, and the appearance of RBs correlates with the disappearance of motile spirochetes [106, 108]. Mistakenly, transformation of the motile form into a RB was considered as a decrease of pathogen viability. However, even though RBs are less motile than spirochetes in a log phase culture, they are able to twitch or move laterally and revert to the active growing reproductive wave-form spirochetes under the favorable conditions [7, 14, 15, 100, 101, 103–107, 109]. In addition to spirochetes from the *B. burgdorferi* (*s.l.*) complex, reversible pleomorphism has been confirmed in other species from the genus *Spirochaeta* [110–113] and strengthened by the discovery of *Spirochaeta coccoides*, a new member of the genus *Spirochaeta* that grows and reproduces in RB forms only [111]. The formation of RBs *in vitro* as a response to exposure to the β-lactam antibiotics commonly recommended for LD treatment was described more than 20 years ago [105]. Persistence of *Borrelia* in hosts after antibiotic treatment has been already confirmed [17, 32, 48, 49, 85]. Atypical cystic forms were also observed in the cerebral cortex of a patient with chronic Lyme neuroborreliosis [101]. *Borrelia* aggregates or biofilms have been detected in skin biopsies isolated from patients who developed rare but typical lymphocytomas after a tick bite [114, 115]. Biofilms, consisting of bacteria embedded in a self-produced polysaccharide matrix, are known to cause chronic infections due to their tolerance to antibiotic treatment and resistance to host serum complement [94, 96, 106, 114, 116].

## Different susceptibility of persisters to antibiotics

Persisters are highly heterogeneous [8, 96–101, 104, 106, 114]. Stress persisters differ from antibiotic persisters of the same *Borrelia* species due to the origin of condition that triggered their transformation. *In vitro* persisters differ from *in vivo* persisters due to the difference in environment in which the spirochetes reside [95]. It is still unknown if the same morphological forms, for example RBs formed in response to antibiotic exposure, are the same as RBs that are present in excess in stationary growth culture [109]. Recent studies confirmed that the susceptibility of heterogeneous persisters to a diverse spectrum of antibiotics is also different [102, 117]. Results of the study of three morphological forms of *Borrelia in vitro* revealed that five antimicrobial drugs commonly used as monotherapy in LD cases (doxycycline, amoxicillin, tigecycline, metronidazole and tinidazole) showed different potential in eradicating spirochetes, round bodies and biofilm-like colonies [102]. While significant killing was shown by all five drugs in the case of replicating spirochetes (85–90%) and round bodies (68–90%), neither one of the studied drugs was able to reduce the spirochete colony formation more than 55%. In the same study, viable spirochetes were detected in 70–85% of biofilm-like colonies [102]. Another study [117] revealed that tolerance to antibiotics is increased by different forms of spirochetes as culture ages, from log phase to stationary phase, from spirochete form to RB, micro-colonies or biofilms. The same study confirmed that the use of multiple drugs (triple combination) is much more effective in eradicating spirochete persisters. The complete eradication of biofilm-like spirochete micro-colonies *in vitro* was confirmed for a combination of daptomycin + doxycycline + cefoperazone, something that has not been achieved before with any single, dual or even triple drug combination [117]. Another option for successful eradication of *Borrelia* persisters *in vitro* is the pulse dosing of antibiotics [18]. While a combination of multiple antibiotics from different classes analyzed in the mentioned study did not improve the killing of persisters, four pulse doses of cetriaxone eradicated all live spirochetes in culture, including persisters [18].

The antibiotic treatment of LD is a real challenge, sometimes with unpredicted outcomes, and is another paradox in the LD portfolio. Although seemingly illogical, multiple frontline antibiotics used to cure early LD are triggering, at much lower concentrations than used in therapy, the transformation of the spiral form of *Borrelia* into varieties of persisting forms [12, 106]. As a result, the *Borrelia* population becomes tolerant to the antibiotics that induced the morphological transformation. A good example of this paradox is doxycycline and amoxicillin, which at high concentrations are capable of eliminating 98% of replicating *Borrelia*, but show poor activity against a stationary phase culture that is rich in persisting forms. Both drugs are ineffective against RBs, the formation of which they induce in culture [12, 102, 105, 106, 108]. It is interesting to note that morphologically changed forms represent spirochete populations with different gene expression profiles, characteristic for the specific antibiotic, doxycycline or amoxicillin, used for *in vitro* culture treatment. It was shown that treatment of *B. burgdorferi* B31 culture for six days with 50 μg/ml of doxycycline or 50 μg/ml of amoxicillin resulted in differential expression of 675 genes in the doxycycline-tolerant population (340 downregulated and 335 upregulated) and 516 genes in the amoxicillin-tolerant population (174 downregulated and 342 upregulated) [12]. Binding to the 30S ribosomal subunit, doxycycline obstructs the protein synthesis that causes the cell envelope defect. Amoxicillin inhibits the synthesis of bacterial cell walls that results in the death of bacteria. A comparison of the pathways of doxycycline- and amoxicillin-induced RBs shows that they share some common features that likely contribute to the enabling of spirochete survival under antibiotic pressure by downregulation of outer membrane lipoprotein gene expression. It is possible that one of the strategies spirochetes use to survive antibiotic treatment is the reduction of drug targets [12].

Amoxicillin-induced RBs have been used as an alternative model to stationary phase culture of *B. burgdorferi* in screening drugs active against persisters *in vitro.* Both models showed different susceptibilities to the same tested drugs. Because the structure of stationary phase culture is more complex, as it is enriched with RBs, micro-colonies and biofilm- like aggregates, its tolerance to tested antibiotics was shown to be higher in comparison to amoxicillin-induced round bodies [109].

## Genetic factors involved in persistence

A genetically homogeneous population of spirochetes can dynamically alter gene expression in response to changing environmental conditions. When external stimuli on a bacterial subpopulation randomly trigger expression of genes that induce a state of dormancy, then a persister subpopulation can emerge. Persistence only occurs in a subpopulation of *Borrelia* cells that faces hostile pressure and is characterized by switching between two phenotypes, i.e. susceptible and persistent. *Borrelia burgdorferi* has been shown to persist when treated with tetracycline antibiotics [49]. Even though the mechanism of persistence for *Borrelia* has not yet been described, studies of bacterial persisters in fungi, parasites or viruses with a number of identified pathways and genes have shed light on the mechanism of persister formation and survival. The identified pathways in bacterial persisters could serve as potential targets for the development of new anti-persister drugs. These include, for example, toxin-antitoxin modules (*hipBA*, *relBE*, *mazEF*, *tisAB*, *mqsR*, *hhA*, *hokA*, *cspD*, *pasT*), stringent response (*relA*, *dksA*), DNA repair or protection (*lexA*, *recA*, *recB*, *xerC*, *xerD*, *dps*), phosphate metabolism (*phoU*), alternative energy production (*sucB*, *ubiF*, *glpD*, *plsB*, *tgs1*), anti-oxidative stress or macromolecule degradation (superoxide dismutase, catalase) or signaling pathways (*comE/comC; tnaA*, *oxyR*, *flu*, *pspBC*) (for review see [95]). As *in vitro* persisters differ from *in vivo* persisters, a drug that can kill all *in vitro* persisters is not guaranteed to do so *in vivo*. Nevertheless, the *in vitro* persisters may share some common features with *in vivo* persisters and *in vitro* persister models should still have significant value in persister studies as surrogates of *in vivo* persisters. Possible overlap between different persisters can be studied using single-cell techniques or RNA sequencing analysis to determine the expression pattern of genes that are differentially regulated by the pathogen as a survival strategy in response to hostile pressure.

## Biofilms

In general, biofilm formation is an example of bacterial adaptation to a changing environment, occurring primarily for four reasons: (i) defense; (ii) colonization; (iii) community; and (iv) the default mode of bacterial growth [118]. In particular, biofilm formation is a stage of microbial development and a part of the mechanism of establishment of chronic bacterial infection [119–121]. More than 40 years have already passed from the first mention of tangled fibers of polysaccharides that extend from the cell surface and form “glycocalyx” around the individual cells or a colony of bacteria. The glycocalyx-mediated adhesion was described as a major determinant in the initiation and progression of the wide spectrum of bacterial diseases, from dental caries to pneumonia [122]. The new description defines biofilms as a microbial-derived sessile community of cells irreversibly attached to a substratum, interface or to each other, embedded in a matrix of extracellular polymeric substances (EPS) secreted by them and exhibit an altered phenotype of growth and gene transcription [119]. Biofilms are complex structures, highly resistant to environmental and therapeutic pressure. The primary polymeric compound of extracellular polymeric substance is alginate, a non-sulfated mucopolysaccharide described as a major biofilm component for multiple bacterial species, including those from the order Spirochaetales, *Leptospira biflexa* and *Treponema denticola* [123, 124] and *Borrelia burgdorferi* [96]. Even though the chemical structure of mature biofilms is diverse and includes phospholipids, polysaccharides, proteins, glycoproteins, embedded calcium and extracellular DNA (eDNA), alginine, calcium and eDNA are considered as a typical markers of biofilms [96]. The compilation of genes, conducted by K. Jefferson, that are required for biofilm formation (the cause genes) and those differentially expressed in an established biofilms (the effect genes) in multiple bacterial species include numerous genes involved in adhesion, quorum sensing, cell wall synthesis, metabolism, stress response division and motility [118]. Biofilms might be formed by a single bacteria species or represent a multi-species community attached to biotic or abiotic surfaces [121]. The physical characteristic of surfaces influence bacterial attachment only to a minor extent, showing that smooth surfaces are colonized with the same success as rough ones [125]. Once attached to the surface bacteria go through a series of changes that are required for adaptation to the life on surface. Extensive production of EPS is a common adaptation step; it protects the biofilms and might result in biocide resistance [126, 127]. The changes of environmental conditions triggers the transition of free-living bacteria to life on a surface that starts with the early stages that includes cell-surface and cell-cell interactions, followed by the development of mature biofilms and the return to a planktonic mode of growth. Those environmental signals vary and might differ among bacterial species, but in general what might influence the biofilms formation is the media composition/nutrition, temperature, pH, osmolarity, iron or oxygen [121]. Surface-attached bacteria are often associated with an increased synthesis of EPS and the development of antibiotic resistance, the feature that makes mature biofilms a serious clinical problem. It is known that EPS prevents the access of antibiotics to bacterial cells in the biofilm using different mechanisms that include physical or chemical diffusion barriers to antibiotic penetration, slow growth of the biofilms, activation of the stress response or appearance of a biofilm-specific phenotype [128].

The mode of biofilm formation of the causative agent of Lyme disease highly resembles that of other bacteria [129]. It has been shown that *Borrelia* is able to form colonies and develop aggregates with characteristic features of biofilms on biotic and abiotic substrates in a static or low-share-force environment. The use of the atomic force microscopy revealed the presence of channel-like structures in *Borrelia* biofilms that have been previously described in other biofilms and presumably serve as the routes of distribution of the oxygen and nutrition [119, 130]. Sapi et al. [96] showed that the protective extracellular substrate of *Borrelia* aggregates is predominantly composed of alginate with calcium and extracellular DNA. However, contrary to the other bacterial species that share the same pathway for alginine production, regulated by clustered *algA*, *algD* and *algE* genes [131], *Borrelia* most probably uses a yet unknown pathway of alginate production as its genome lacks the genes homologous to that mentioned above. An analysis of the key components of *Borrelia* biofilm formation highlighted the impact of RpoN-RpoS alternative sigma factor pathways that are involved in the bacterial response to the environmental stresses and are responsible for sensing environmental stimuli [132]. The RpoN-RpoS signal-transduction pathway secures the successful transmission of the spirochete from tick to host, regulating the expression of over 100 genes involved in the infectious cycle, survival and stress response of *Borrelia* [133, 134]. Another regulatory pathway vital for *Borrelia* biofilm formation is the quorum sensing system, that by autoinducers triggers the population-wide differential regulation of genes involved in biofilm formation [135–137].

*Borrelia* biofilms have already been described in the midgut of infected tick nymphs during blood-feeding [5]. Using confocal and epifluorescence microscopy, the authors confirmed that during the blood-feeding *Borrelia* spirochetes progress through the nymphal midgut as epithelial cell-associated networks of non-motile organisms. *Borrelia* biofilms have also been detected in human infected skin [114]. The reoccurrence of the disease, the ability of spirochetes to evade the host immune system and the ability to resist antibiotic treatment strongly supports the existence of biofilm-like protective structures in infected patients.

## Conclusions

The list of antibiotics recommended for LD treatment at the early stages of infection is expanding [12, 95, 96, 102, 108, 109, 114]. Current antibiotics are efficient in killing the growing replicative form of spirochetes, but they have rather insufficient activity against non-growing persistent forms. It has been confirmed that monotherapy of *Borrelia* infection with β-lactam, tetracycline, fluoroquinolone, sulfonamide, macrolide, lipopeptide, glycopeptides, aminoglycoside or antitumor antibiotics are not adequate. Such treatment fails to eliminate spirochetes in *in vitro* culture and leaves viable and effective persisters in treated vertebrates, including humans [12, 17, 18, 49, 85, 106, 108, 109, 117]. The obvious need in antibiotics with strong anti-persister activity has led to the identification of drugs that act differently from current LD antibiotics [117]. However, even anti-persister drugs such as daptomycin, clofazimine or daunomycin cannot kill different persister forms such as cysts, round bodies, micro-colonies or biofilms alone. A combination of two or three drugs from different classes of antibiotics with different mechanisms of action along with the use of sulfa drugs shows significant improvement in eliminating multiple persisters’ forms *in vitro*. Pulse dosing treatment shows great potential for eradicating persisters and seems to be a promising scheme for LD treatment. The interest in identifying alternative drug candidates with a high activity against multiple persister forms is growing. The other known “agent”, comparable by its strength to triple-combination antibiotics treatment and efficient in eliminating log phase spirochetes as well as reducing persisters (by 94%), is *Stevia rebaudiana*, the plant widely known as honey leaf or sweet leaf [138]. Most probably the killing power of this “agent”, which is the whole leaf extract of the plant, is the result of the synergy of multiple natural compounds not yet identified. Recently, the screening of wide set of essential oils revealed candidates with an even stronger anti-persister activity than was described for some anti-persister drugs. For example, oregano oil and its active component carvacrol, cinnamon bark or clove bud were more efficient against the stationary phase and biofilms of Lyme disease spirochete than 40 µM daptomycin. For comparison, 0.05% cinnamaldehyde, the active component of cinnamon bark essential oil, sterilized the LD spirochete at stationary phase and garlic essential oil was successful in killing of all forms of *Borrelia* at a concentration of 0.05%. These results were confirmed by the absence of bacterial regrowth after 21 days of subculture and correspond to results obtained by culture treatment with 5 µg/ml of triple-drugs daptomycin + doxycycline + cefuroxime [139, 140]. The majority of published results, dealing with promising or fairly successful anti-*Borrelia* drugs or their combinations, were obtained *in vitro*. There is no doubt that their effect on spirochetes *in vivo* will be different, may be unexpected, and definitely unpredictable. Evaluating the elimination capability of anti-*Borrelia* drugs or finding alternative candidates against multiple non-replicating forms of LD spirochetes should be taken to another level. Until the same experiments are conducted *in vivo*, involving multiple laboratory animal models, concerns about successful LD therapy protocol will remain at the discussion or speculation level. The success of LD therapy depends on how early antibiotic treatment is started. The question is how early is early enough to start antibiotic treatment in order to assure elimination of the replicating forms of spirochetes in an infected host, if a 7–10 day-old *in vitro* culture already contains multiple persistent forms of the pathogen [109]. If the signature marker of Lyme disease, erythema migrans, does not develop in a person after a tick bite, antibiotic treatment will not be initiated immediately. How much time might the motile form of bacteria need to change its morphology or to hide in vertebrate host tissues *in vivo* when such changes triggered by an unfriendly environment *in vitro* can occur in minutes or hours? Even if antibiotic monotherapy is started shortly after exposure to a tick, the LD treatment paradox could occur anyway: frontline LD antibiotics can trigger the establishment of a persister subpopulation *in vivo* as it has been proven by the confirmation of chronic infection in multiple animal species and humans [23, 48, 49, 85, 106, 114]. Atypical dormant spirochete forms, persisters, survive in a *Borrelia*-infected host for years, regardless of antibiotic treatment. The recurrence of LD likely happens because persisters may convert back into motile replicating infective forms under favorable growth conditions. The treatment of LD requires knowledge of its history and *Borrelia* pleomorphism in its natural environment [106]. The theory that chronic spirochete infections in humans are examples of symbioses that developed between the host and pathogen over time has been proposed for LD [106]. The human-*Borrelia* interaction represents co-evolved spirochete-tick-host relationships highly integrated on genetic, metabolic and behavioral levels. From this point of view LD is a manifestation of long-lasting genetically integrated symbioses, and the disease symptoms are the expression of symbiogenesis to which many aspects of the host immune system respond [106]. Symbiogenesis means cooperation between species in order to increase their survival. While the benefit of symbiosis to the spirochete is obvious, what is the benefit of this symbiosis to the host? The successful persistence of spirochetes within the host is known to depend on their ability to use the host regulatory proteins to avoid recognition and eradication by its complement. Inability to understand chronic LD as ancient co-evolved host-pathogen symbioses might lead to misdiagnoses and insufficient therapies. The probability that chronic LD arises from a persisting infection is real. Regardless of the cause of chronic LD, i.e. persisting forms hidden in biofilms [49, 96], cell-wall deficient forms [31], or round bodies [15], the *in vivo* treatment options for all of them are very limited. Addressing this problem requires a new comprehensive examination of the complex and controversial subject called chronic Lyme disease.

## Data Availability

Not applicable.

## Abbreviations

LD: Lyme diseases
RB: round bodies
EPS: extracellular polymeric substances
eDNA: extracellular DNA
